# Don’t touch that dial: Psychological reactance, transparency, and user acceptance of smart thermostat setting changes

**DOI:** 10.1371/journal.pone.0289017

**Published:** 2023-07-24

**Authors:** Matthew Heatherly, D. A. Baker, Casey Canfield

**Affiliations:** 1 Department of Engineering Management & Systems Engineering, Missouri University of Science & Technology, Rolla, MO, United States of America; 2 Department of Psychological Science, Missouri University of Science & Technology, Rolla, MO, United States of America; National Taiwan University, TAIWAN

## Abstract

Automation inherently removes a certain amount of user control. If perceived as a loss of freedom, users may experience psychological reactance, which is a motivational state that can lead a person to engage in behaviors to reassert their freedom. In an online experiment, participants set up and communicated with a hypothetical smart thermostat. Participants read notifications about a change in the thermostat’s setting. Phrasing of notifications was altered across three dimensions: strength of authoritative language, deviation of temperature change from preferences, and whether or not the reason for the change was transparent. Authoritative language, temperatures outside the user’s preferences, and lack of transparency induced significantly higher levels of reactance. However, when the system presented a temperature change outside of the user’s preferences, reactance was mitigated and user acceptance was higher if the thermostat’s operations were transparent. Providing justification may be less likely to induce psychological reactance and increase user acceptance. This supports efforts to use behavioral approaches, such as demand response, to increase sustainability and limit the impacts of climate change.

## Introduction

Global energy consumption is growing faster than the human population with each person consuming more energy each passing year [1]. Residential energy usage accounted for about 39% of energy consumption in all U.S. sectors in 2021 [2]. With residential consumption, the average American spends approximately 55% of their total energy expenditure on heating and air conditioning [3]. An ever-growing consumption of electricity results in the increasing production of greenhouse gasses from burning fossil fuels, leading to negative sustainability outcomes [4]. These greenhouse gasses have been the leading cause driving decades-long global warming trends [4]. Exacerbating the issue, increasing extreme weather events and rising global temperatures create an even greater demand for energy to heat and cool homes. The Energy Information Administration predicts air conditioning demand will triple by 2050 [5].

To encourage reduced electricity usage and sustainable consumption, a variety of strategies have been used (e.g., see [6]). One strategy that has been implemented in several areas across the United States is referred to as demand response. Demand response, where the price of electricity fluctuates to act as a price signal, incentivizes reducing or shifting energy usage during peak periods. However, the implementation of demand response systems comes with a few challenges, including scalability, security, and user acceptance [7]. One potential solution to the difficulties of implementing demand response systems is through the use of automated smart home technology. However, these automated systems must be persuasive without inducing negative reactions, which is a complex endeavor [8].

Smart energy management systems monitor energy consumption and perform energy conservation behaviors according to their programming [9, 10]. Smart energy management systems work well with demand response systems as they can be operated directly by the utility company, which may support a stronger relationship between utilities and consumers [11–13]. Current research suggests that cities may be able to approach net zero via the reduction of carbon emissions within urban infrastructure and by promoting accounting and reporting practices to track greenhouse gases [14]. In the corporate and industrial sectors, environmental management systems and technologies have been effective for enhancing corporate environmental sustainability [15].

Previous research has shown that user interface design can be as critical to user acceptance of smart thermostats as the functionality itself [16, 17]. A key design challenge for achieving user acceptance is providing users with the appropriate level of information about the system’s actions so as to imply control and choice [18]. Yang & Newman (2012) found that a critical contributing factor leading to disuse of the Nest smart home thermostat was uncertainty about what the system was doing [19]. Pisharoty et al. (2015) found that a smart thermostat design that learned user habits and then presented three possible schedules for a user to choose from reduced energy usage by an estimated 4.7% over manual programming and 12.4% over Nest (which does not present any options) [20]. However, this schedule was set only once at the beginning of the 3-month study and did not examine how users would respond to changes in the thermostat settings due to, for example, demand issues, current weather conditions, or changes in occupancy.

Reactive systems can further energy savings by using dynamic sensor data, network data, and machine learning to make temporary adjustments to a preset schedule. Yang et al. (2014) argue that maximizing energy savings with reactive smart thermostats will require the system “to push information, requests, and suggestions to the user” rather than expect users to be aware of these dynamic economic and environmental conditions and then respond accordingly [21]. The user interface for such a system can either inform users of a thermostat change that is about to happen without user intervention (i.e., it will change), that they can institute themselves (i.e., you could/should change), or that has occurred already (i.e., it has changed).

The automation of smart energy management systems makes it a convenient option for people wanting to conserve energy. However, too much automation can reduce user acceptance as people still want to have a certain degree of control over their smart technology [22]. In this case, there is a risk of inducing psychological reactance in the user. While research has focused on optimizing energy management systems in terms of improving Internet of Thing (IoT) features [22, 23], computational techniques [24], security [25], artificial intelligence [26–28], and other technical aspects [29], less investigation has gone into the underlying psychological mechanisms involved with appropriately using and communicating with said systems. Due to the complex nature of implementing sustainability measures, it is becoming increasingly important to explore multidisciplinary solutions to bridge the gap between green technology and consumer behavior [30]. This can create more effective systems to tackle the energy and sustainability crisis as well as other large-scale socio-ecological problems.

### Psychological reactance

First described by Brehm (1989), psychological reactance is a motivational state a person may experience when they feel their autonomy is threatened. The threat can be direct or implied [31]. For example, a parent telling their child to wash the dishes directly threatens the child’s ability to choose whether they do the dishes or not, while one sibling asking if they can use the family car for the evening indirectly threatens the other sibling’s freedom to use the vehicle themselves that evening. This motivational state can lead a person to engage in behaviors to reassert the threatened autonomy [31]. These behaviors may be something a person previously had no interest in engaging in and may even be counter-attitudinal–that is they previously did not want to engage in the behavior but do so anyway when the freedom to choose is threatened. For example, if the sibling who previously had no interest in using the family car was initially looking forward to a quiet evening at home, then suddenly feels motivated to do car-related activities when they hear that the other sibling might be monopolizing the vehicle.

Furthermore, even in scenarios where a person ostensibly receives a benefit from a persuasive message, the perception of a threat to their autonomy can induce reactance. This is demonstrated in findings by Reinhardt & Rossmann (2021) where participants experienced reactance when presented messages containing persuasive language concerning receiving a vaccine [32]. While the administration of vaccines has the intention of benefiting people, an individual may experience reactance (and refuse to get a vaccine) when they believe their autonomy over the decision to get the vaccine is threatened. In the context of smart energy management, a system that induces psychological reactance might lead a person to override an energy saving thermostat setting merely to reassert freedom and control, even if the change would have had no immediate effect on their thermal comfort, leading essentially to a backfire effect for both comfort and energy savings.

Reactance is not induced with every loss or threat to autonomy. Rather, research suggests a number of factors influence whether it occurs or not, including (1) authoritative language, (2) alignment with user goals, (3) perceived legitimacy and permanence, and (4) social agency. For example, highly authoritative language is more likely to induce reactance than mild, polite language [31, 33, 34]. Miller et al. (2007) found that when manipulating promotional health messages, using high controlling language such as “have to”, “must”, and “should” resulted in participants experiencing more reactance, while low controlling language such as “could” and “might” caused less reactance [35]. This is consistent with findings from Roubroeks et al. (2010) where participants experienced more reactance when a robot washing machine used high threatening language (e.g., “You have to set the temperature to 40°C”) during a direct interaction [36]. Furthermore, a study by Babel et al. (2021) indicated participants experienced uncooperative behavior, reactance, and even fear when working with robots using threatening or commanding conflict resolution strategies [37]. A key design challenge for home automation of energy management is balancing users’ dynamic preferences for thermal comfort with actual energy savings. This is complicated by reactance. That is, users will likely want to be notified about impending thermostat setting changes because it could differentially affect their thermal comfort depending on the type of activities they are engaged in at the time. However, the way the notification is worded could affect a user’s motivation to accept or reject those changes even more than the actual setting change itself. Therefore, we propose hypothesis 1:

*H1: Highly authoritative language will induce reactance and reduce user acceptance*.

While language is important, Roubroeks et al. (2010) also found that when their persuasive robot washing machine goals aligned with the user’s goal (e.g., saving energy or washing the clothes more thoroughly), the user was less likely to experience reactance [36]. For residential smart energy management, there may be competing user goals to manage. Therefore, beliefs and attitudes about what thermostat setting range is ideal for achieving personal comfort goals could influence whether deviations from that range are perceived as an impingement on freedom. On the other hand, users may have energy saving goals or budget goals that are, at times, in conflict with comfort goals. As such, smart energy management systems are being designed to address and maximize a number of user values at once [38] and may seek to adjust the thermostat to a temperature outside a user’s normal setting range in order to do so (or simply to maintain a subjective thermal comfort goal under extreme weather conditions). Research has not examined whether small deviations from a user’s typical settings (for example, 2 degrees) will induce reactance, nor whether this is mitigated if such a deviation helps achieve competing goals. Therefore, we propose hypothesis 2:

*H2: Deviating from user-preferred thermostat settings will induce reactance and reduce user acceptance*.

Reactance may also be affected by the perceived legitimacy of a potential threat to freedom and the individual’s ability to rationalize that threat. Although reactance literature finds that both legitimate and illegitimate requests may induce reactance, there is some evidence that illegitimate requests evoke a stronger immediate response than do legitimate requests [39, 40]. Further, Brehm (1989) argued that reactance is unlikely to occur if a person is made aware that the threat or loss is temporary and exceptional [31]. This is supported by research from Ehrenbrink et al. (2016) who found that when a voice-controlled smart TV committed an error, reactance was lower when the error was accompanied by information showing that the system had misinterpreted what the user just said, as compared to an error that occurred without this information [41]. This suggests reactance may be mitigated by providing a justification or clarifying the nature of a threat to one’s autonomy. While reactance is considered an irrational response [31], introducing an element of rationality could reduce the effects of the state. Therefore, we propose hypothesis 3:

*H3: Removing a justification will induce reactance and reduce user acceptance*.

Finally, reactance is more likely to occur when the request is perceived as coming from an agent with some degree of social agency [42], which can be cued in a number of ways such as a human-like face, facial expressions, and speech [43]. For example, Ghazali et al. (2018) found that when participants received high-control advice, reactance was higher when interacting with a robot and higher still when robots employed social cues, such as head movement, facial expression, and tone of speech output [43].

### Research aims

Reduced user acceptance of smart home energy management systems could stand in the way of increasing energy efficiency. Certain types of notifications could create a backfire effect where rather than improving user acceptance, users override the system to reassert their perceived loss of freedom (i.e., experience reactance). This is distinct from overriding the system for comfort or cost savings. To date, few studies have explicitly examined the interactions between factors that affect reactance in the context of energy saving behavior. The primary contributions of this study are (a) developing an experimental paradigm where reactance can be observed and manipulated in an energy context, (b) replicating findings from other domains, and (c) finding an indirect relationship between reactance and behavioral intentions that is influenced by the presence of a justification. This study demonstrates that in the energy context some messages do induce reactance (i.e., authoritative language, suggesting a temperature incongruent with user preferences) as well as the effectiveness of a potential solution (i.e., including a justification for the temperature change) to increase compliance.

In this study, users interact with a hypothetical smart thermostat. This online experiment employs principles of design fiction, a practice of exploring hypothetical technologies and realities via creative design choices [44–46]. We experimentally manipulate authoritative language (Language: Might, Has, Will, Must), alignment with user goals (Temperature: Congruent, Incongruent), and perceived legitimacy and permanence (Justification: Transparent, Intransparent) in a between-subject design. In addition to testing the pre-planned hypotheses described above with ANCOVAs, we conduct exploratory analysis on interaction and mediation effects. The findings are discussed in terms of their implications for smart energy management system design, sustainability, and other green behaviors as well as broader implications for human-machine interaction. The remainder of this paper describes the methods (section 2), results (section 3), discussion (section 4), and conclusion (section 5).

## Materials and methods

### Participants

Participants were recruited through Prolific, an online survey platform that offers a diverse participant pool [47]. All participants were over 18 years old and resided in the US. Each participant was compensated $2.50. In total, 500 participants were recruited with 51% being male, 46% female, 2% non-conforming, and 1% did not specify. Sixty-three percent were White, 19% Asian, 6% Black/African American, 5% Hispanic, less than 1% were Native American / Alaskan Native, and less than 1% reported “Other”. The mean age of the sample was 33 years old (Standard Deviation (SD) = 11) and ranged from 18–78 years old. In addition, 89% reported having at least some college education. The participants were recruited from 48 different states and 92% reported using thermostats in their homes. All participants passed the attention check and minimum completion time requirements; therefore, all recruited participants were included in the final analysis.

### Design

This study experimentally manipulates a message notification (ostensibly sent from a smart thermostat) across three dimensions using a 4 (Language: Might, Has, Will, Must) x 2 (Temperature: Congruent, Incongruent) x 2 (Justification: Transparent, Intransparent) between-subjects factorial design. Participants were randomly assigned to each condition. All materials, data, and R code are available at Open Science Framework https://osf.io/zufmt/. The study was approved by the Institutional Review Board of the University of Missouri System and written consent was obtained.

### Variables

The Language variable altered the degree of authoritativeness, or threat to autonomy, used in the message notification. Based on previous literature, the Might and Has conditions are included as a low threat, while Will and Must represent higher threat [35, 48]. The Has condition is included as a low threat based on research showing that when a freedom is clearly already lost and therefore energy must be expended to restore the freedom, reactance may not be experienced, or may quickly disappear [49].

The Temperature variable altered the suggested temperature setting contained in the message notification. To create this manipulation, participants were first asked to indicate their usual thermostat setting range for a typical cold winter day (see procedure). Those assigned to the Congruent condition saw a setting within their range (the midpoint between their stated low and high setting, rounded up to the nearest whole number). Those assigned to the Incongruent condition saw a setting that was two degrees colder than their stated low temperature preference.

The Justification variable was experimentally manipulated by including weather information for some participants (Transparent), while not including it for others (Intransparent). Participants in the Transparent condition received the message “It is currently 50°F with humidity of 45% and wind speeds of 6 mph.”, while those in the Intransparent condition did not receive this message.

The primary outcome variables measured three aspects of state reactance. State Reactance is the immediate response to a threat to autonomy and is thought to be driven by two intertwined processes: a cognitive response, characterized by the formation of negative thoughts (Negative Cognitions) and an affective response, characterized by feelings and expressions of anger (Anger) [39]. Threat to Freedom is the perceived threat to one’s autonomy and is indicative of an individual experiencing state reactance.

Negative cognitions were measured using a single item from Dillard et al. (2018), which asks “Overall, I would describe my thoughts toward the thermostat as:” [50]. Responses were indicated using a 5-point Likert scale, where 1 = “Extremely Positive” and 5 = “Extremely Negative”.

Anger is the emotional response felt by the participant and is distinct from negative cognitions, for example, someone can think that something is bad (negative cognition) but they may not be angry about it (emotional response). Anger was measured using a four-item scale from Dillard & Shen (2005) [39]. The scale contained items such as “The amount of anger I feel after the above message is:” and “The amount of annoyance I feel after seeing the above message is:”. Responses were indicated using a 5-point Likert scale, where 1 = “None at all” to 5 = “A great deal”.

Threat to Freedom measures the degree to which an individual perceives their autonomy as being threatened. It was measured using a four-item scale from Dillard & Shen (2005) [39]. The scale included items such as “The thermostat tried to make a decision for me” and “The thermostat threatened my freedom to choose.” Again, responses were indicated using a 5-point Likert scale, this time with 1 = “Strongly Disagree” to 5 = “Strongly Agree”. For Anger and Threat to Freedom, the scores were calculated by taking the average for each 4-item scale.

In addition, a measure of user acceptance of the thermostat setting change (Behavioral Intention) was included. This was a single item which asked, “How likely are you to accept the new temperature: [TEMP]” where [TEMP] represents the temperature that was presented to the participant, which was based on their specified range and their randomly assigned Temperature condition. Responses were indicated using a 5-point Likert scale where 1 = “Extremely Unlikely” and 5 = “Extremely Likely”,

Two potential covariates were assessed. First, the importance that a threatened freedom holds can also influence whether (and to what degree) reactance is experienced, where threats to an important freedom can induce greater reactance than threats to a less important freedom [51]. To capture this, when participants were asked to indicate their preferred thermostat settings, they were also asked how strongly they felt about keeping the thermostat setting within that specified range (referred to as Strength of Preference). Second, trait reactance is the predisposition a person has to experiencing state reactance [52] and is often included as a covariate when examining state reactance. Individuals with higher trait reactance are more likely to experience state reactance [53]. To measure trait reactance (Trait Reactance), we used the 11-item Hong Psychological Reactance Scale [54].

### Procedure

**Fig 1:**
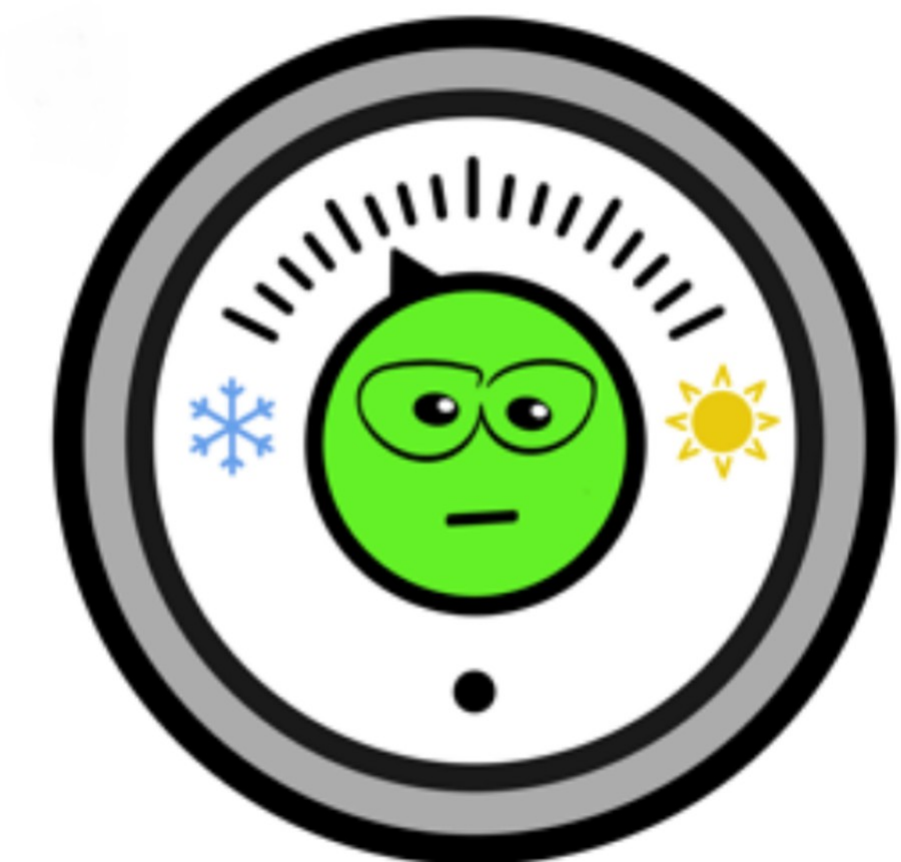
Image of the smart thermostat, SEM. SEM is depicted in the thermostat description of the survey.

First, participants reported whether they used a thermostat at home. If so, they were asked to report their highest and lowest thermostat setting preferences for typical cold winter days, and to indicate how important their temperature preferences were to them. Participants who did not use a thermostat were asked to answer the questions as if they were to use one. Next, participants read a short definition of smart technology and a description of a hypothetical smart thermostat, named SEM (Smart Energy Management), that could make adjustments based on “your setting preferences as well as past utility data from similar homes, external environmental conditions, and scientific data about thermal comfort.” With this description of SEM, an image of a humanoid character with a neutral facial expression was provided (see Fig 1). Participants were then asked to imagine they received one of these thermostats from their utility company and were now setting it up for use in their own home. As mentioned, reactance may increase when system and user goals are not aligned [36]. Since we sought to examine only the effects of language, temperature and justification, rather than the effects of goal alignment (or misalignment), we first allowed each participant to choose a system “mode” as a control measure. This was to create the perception for each participant that the system’s priorities were aligned with theirs. They chose one of two operating modes, “Comfort mode” or “Green mode.” Comfort mode was described as prioritizing user comfort, while Green mode was described as prioritizing energy conservation. Then, to assess attention, participants were asked three multiple-choice questions about what they read.

**Fig 2:**
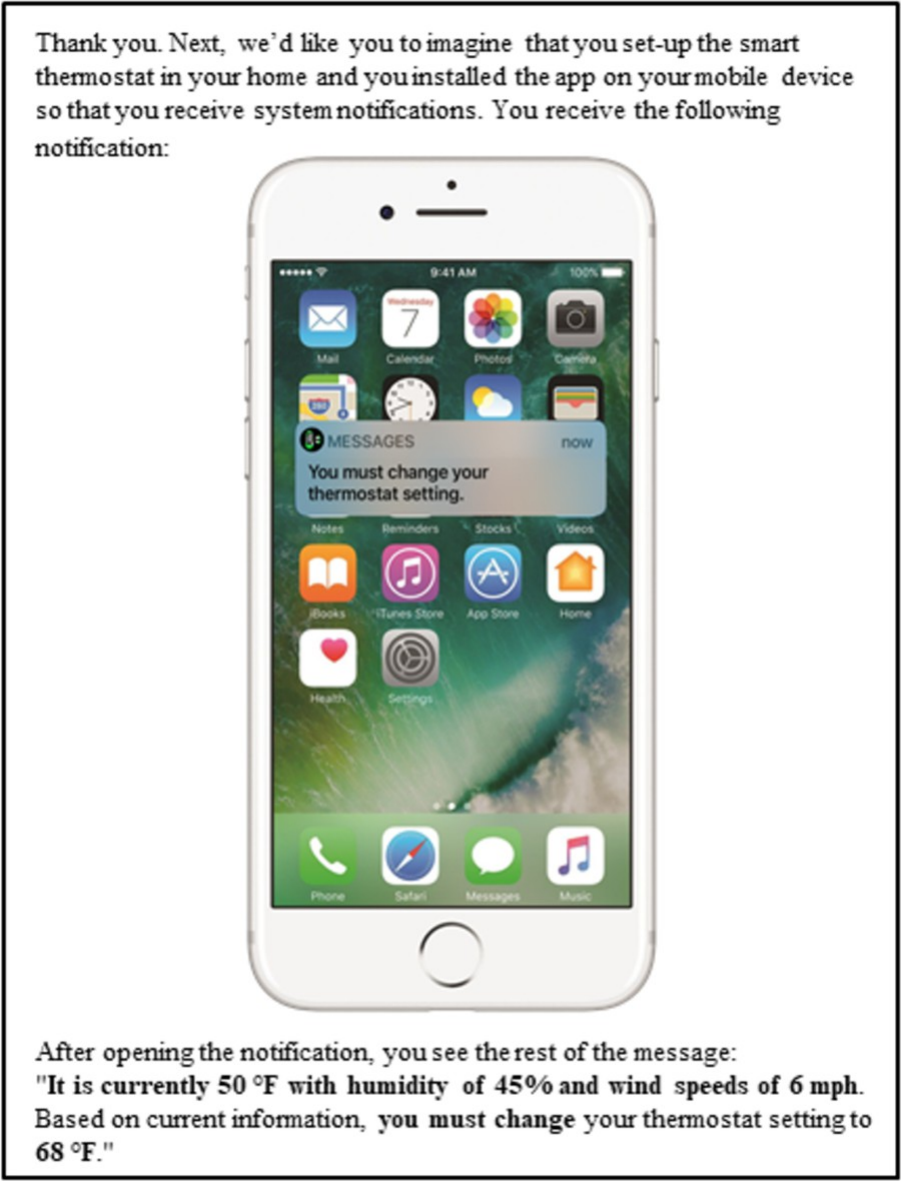
Recreation of image and text manipulation. This is for a participant who indicated a low temperature setting of 70°F, and was assigned to the condition Language = Must, Temperature = Incongruent, Justification = Transparent. (Image and background photo, credit D.A.B.).

Next, participants were asked to imagine that the system was now up and running and that they just received a system notification via their smartphone. They were then presented with an image of a smart phone showing an alert notification, which contained the experimental manipulation for Language. The image was accompanied by text that repeated the verbal content of the notification and provided the Temperature and Justification manipulations. The phone notification image was included to increase authenticity and representativeness, but due to readability concerns, the full message was provided in text (see Fig 2). Immediately after presenting the notification and text, participants completed the state reactance measures. Following the state reactance measures, participants’ behavioral intention was assessed. Participants then saw a second message notification that repeated their assigned language and justification manipulation but was always an Incongruent temperature, which is not analyzed here. Lastly, participants completed the Hong Psychological Reactance Scale and were asked to provide demographic information including age, gender, race, and education.

## Results

### Effect on reactance

Means and standard deviations for the three state reactance measures are reported in Table 1 by condition. Negative Cognitions had a skewness of 0.37, indicating an approximately symmetric distribution. Anger had high internal validity (Cronbach’s ɑ = 0.94) across the four items and as such, the four items were averaged to obtain a single Anger score. The Anger score had a high skewness of 1.8, indicating that most participants felt little anger across all groups. The four Threat to Freedom items had acceptable internal validity (Cronbach’s ɑ = 0.80) and were averaged to create a single score. The Threat to Freedom score had a moderate skewness of 0.59. Table 2 shows the Spearman Correlations between the state reactance measures and covariates. Given the large number of comparisons, it is most appropriate to use ɑ = .001 to evaluate significance. This reduces the probability of a false positive to < 5%. For reference, significance at ɑ = .01 and ɑ = .05 are also provided and described as weak evidence.

**Table 1:** Descriptive statistics for state reactance by condition.

Notification Type				Negative Cognitions	Anger	Threat to Freedom
Language	Temperature	Justification	N	M (SD)	M (SD)	M (SD)
Might	Con	Trans	30	2.30 (0.70)	1.16 (0.30)	2.17 (0.57)
		Intrans	29	2.48 (0.78)	1.44 (0.66)	2.22 (0.80)
	Incon	Trans	43	2.53 (0.80)	1.37 (0.50)	2.09 (0.73)
		Intrans	23	2.78 (0.95)	1.55 (0.92)	2.50 (0.93)
Has	Con	Trans	29	2.10 (0.77)	1.09 (0.27)	2.37 (0.68)
		Intrans	35	2.54 (0.70)	1.54 (1.08)	2.36 (0.85)
	Incon	Trans	38	2.42 (0.92)	1.59 (0.90)	2.57 (0.72)
		Intrans	22	2.95 (0.90)	1.82 (0.81)	2.51 (0.85)
Will	Con	Trans	23	2.22 (0.80)	1.35 (0.60)	2.39 (0.35)
		Intrans	34	2.41 (0.86)	1.47 (0.62)	2.57 (0.79)
	Incon	Trans	40	2.43 (0.93)	1.51 (0.84)	2.49 (0.71)
		Intrans	29	3.14 (0.95)	2.26 (1.12)	3.09 (0.89)
Must	Con	Trans	25	2.60 (0.91)	1.66 (0.72)	2.66 (0.76)
		Intrans	44	2.89 (1.02)	1.83 (0.90)	2.97 (0.90)
	Incon	Trans	24	2.92 (1.14)	2.33 (1.15)	3.22 (0.95)
		Intrans	32	3.47 (1.05)	2.26 (1.17)	3.26 (1.02)

**Table 2:** Spearman correlations for dependent variables, covariates, and controls.

Measure	1	2	3	4	5	6	7	8	9
**Dependent Variables**									
1. Negative Cognitions	-								
2. Anger	0.56*	-							
3. Threat to Freedom	0.45*	0.59*	-						
4. Behavioral Intent	-0.61*	-0.55*	-0.36*	-					
**Covariates / Controls**									
5. Trait Reactance	0.12^	0.28*	0.30*	-0.16*	-				
6. Strength of Preference	-0.01	0.10^+^	0.05	-0.09	0.02	-			
7. Mode (Green)	-0.08	-0.13^	-0.17*	0.14^	-0.13^	-0.17*	-		
8. Education (College)	0.03	0.08	0.14^	-0.02	0.02	0.03	-0.04	-	
9. Gender (Male)	0.02	0.04	0.05	-0.01	0.07	-0.04	0.01	-0.09	-
10. Age	0.08	0.14^	0.09	-0.12	-0.01	0.20*	-0.11^+^	0.18*	-0.06

^+^ *p* < .05

^^^ *p* < .01

* *p* < .001

A series of three-way ANCOVAs were conducted using Language, Temperature, and Justification as the independent variables and each of the three reactance measures as dependent variables. As shown in Table 2, Trait Reactance was significantly positively correlated with Anger and Threat to Freedom. Trait Reactance and Strength of Preference were included as covariates and we also controlled for gender, education, age, and the thermostat mode chosen by the participant. The data satisfied the assumptions required to perform ANCOVAs (i.e., normality, homogeneity of variances, homogeneity of regression slopes, and linearity). Although the group sizes were not equal (see Table 1), research has suggested that as long as the assumption of equal slopes applies, an ANCOVA can be justified under random allocation [55].

**Fig 3:**
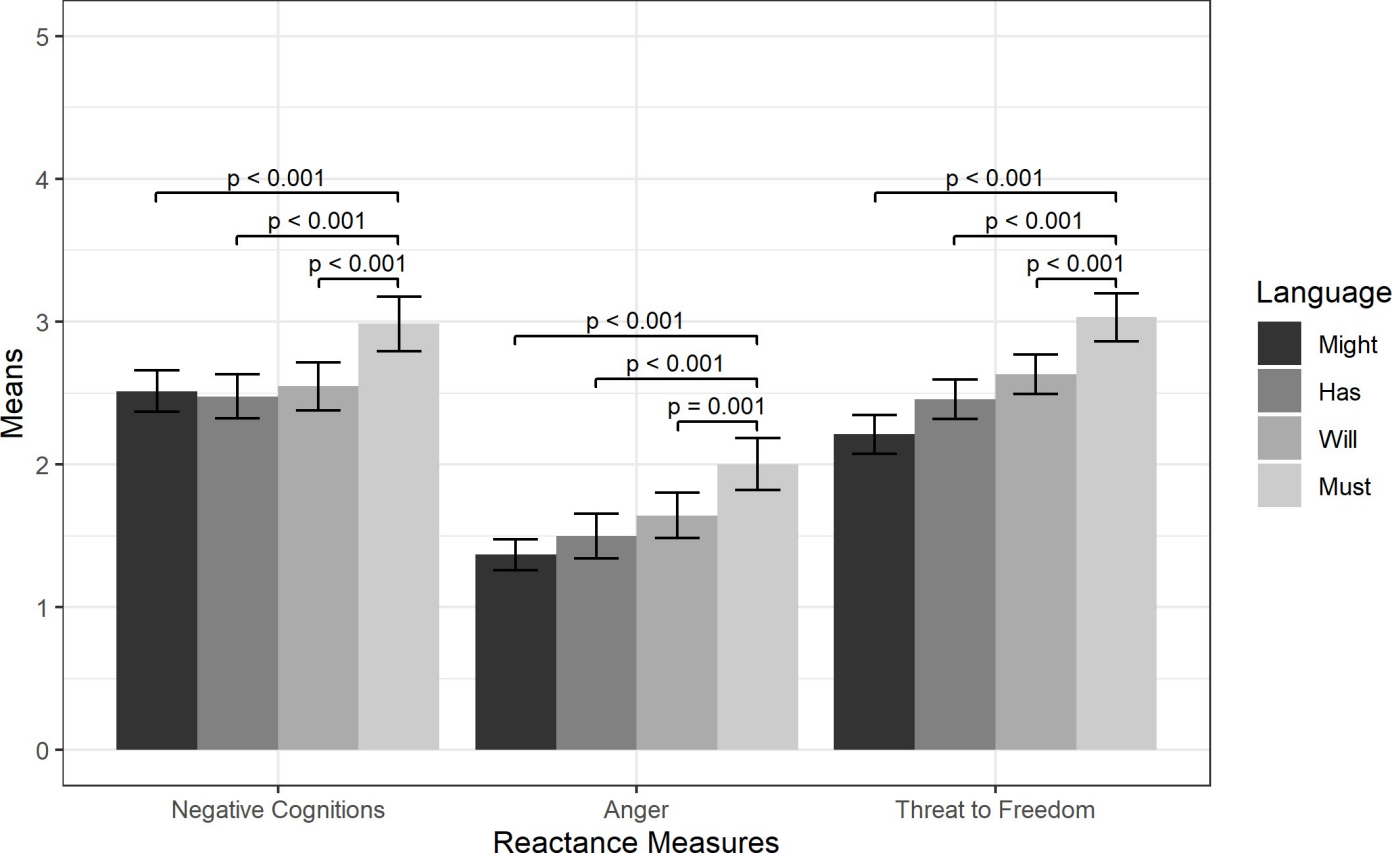
Reactance measure means by Language condition. Results from Tukey post-hoc tests are shown with +/- 2 standard error bars.

We found a main effect of Language on Negative Cognitions, Anger, and Threat to Freedom. A Tukey post-hoc test indicated that for Negative Cognitions, Must (M = 2.98, SD = 1.06) was significantly higher than Might (M = 2.51, SD = 0.81, p < 0.001), Has (M = 2.48, SD = 0.86, p < 0.001), and Will (M = 2.55, SD = 0.94, p < 0.001), but these latter three did not differ from one another. This pattern was also true for Anger, where Must (M = 2.00, SD = 1.02) was significantly higher than Might (M = 1.37, SD = 0.61, p < 0.001), Has (M = 1.50, SD = 0.87, p < 0.001), and Will (M = 1.64, SD = 0.88, p = 0.001), but these three groups did not differ significantly from one another. Similarly for Threat to Freedom, Must (M = 3.03, SD = 0.93) was significantly higher than Might (M = 2.21, SD = 0.76, p < 0.001), Has (M = 2.46, SD = 0.77, p < 0.001), and Will (M = 2.63, SD = 0.77, p < 0.001) with the latter three conditions not differing from one another. These results are displayed in Fig 3.

**Fig 4:**
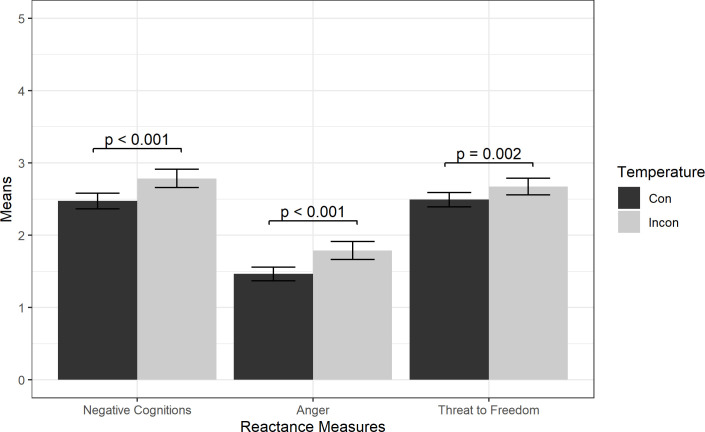
Reactance measure means by Temperature condition. Results from ANCOVAs are shown with +/- 2 standard error bars.

There was also a main effect of Temperature on Negative Cognitions and Anger. As shown in Fig 4, the ANCOVA indicated the Incongruent condition induced significantly higher reactance scores than the Congruent condition for Negative Cognitions (M = 2.78, SD = 1.00 vs. M = 2.47, SD = 0.86, p < 0.001) Anger (M = 1.79, SD = 0.98 vs. M = 1.47, SD = 0.75, p < 0.001). There was weaker evidence of a main effect of Temperature on Threat to Freedom (M = 2.67, SD = 0.92 vs. M = 2.49, SD = 0.79, p = 0.001).

**Fig 5:**
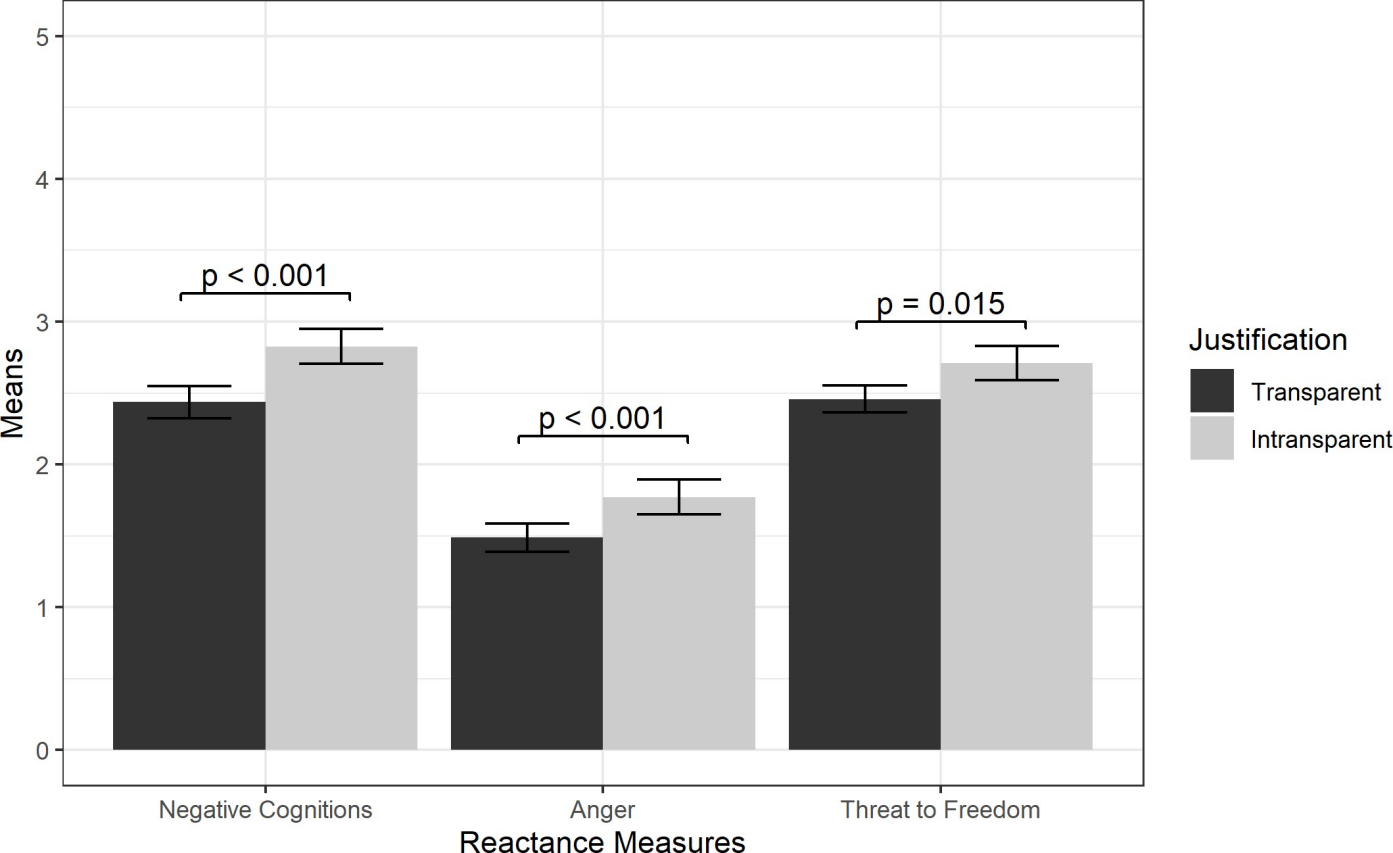
Reactance measure means by Justification condition. Results from ANCOVAs are shown with +/- 2 standard error bars.

Finally, there was a main effect of Justification on Negative Cognitions and Anger. As shown in Fig 5, the Intransparent condition induced significantly higher reactance scores than the Transparent condition for Negative Cognitions (M = 2.83, SD = 0.96 vs. M = 2.44, SD = 0.89) and Anger (M = 1.77, SD = 0.97 vs. M = 1.49, SD = 0.78). There was weaker evidence of a main effect of Justification on Threat to Freedom (M = 2.71, SD = 0.94 vs. M = 2.46, SD = 0.76). ANCOVA results of all main effects are shown in Table 3. Interaction effects, which were insignificant, are shown in S1 Table.

**Table 3:** ANCOVA results for main effects on reactance. Interactions are reported in S1 Table.

Measure	Reactance Measure	SS^a^	df	F	P	*η*_p_^2^
Language	Negative Cognitions	17.19	(3, 497)	7.35	< 0.001***	0.045
	Anger	26.62	(3, 497)	15.12	< 0.001***	0.082
	Threat to Freedom	38.34	(3, 497)	23.41	< 0.001***	0.133
Temperature	Negative Cognitions	16.06	(1, 499)	20.59	< 0.001***	0.044
	Anger	14.69	(1, 499)	25.02	< 0.001***	0.053
	Threat to Freedom	5.34	(1, 499)	9.79	0.002**	0.020
Justification	Negative Cognitions	17.45	(1, 499)	22.37	< 0.001***	0.045
	Anger	5.51	(1, 499)	9.38	< 0.001***	0.021
	Threat to Freedom	3.23	(1, 499)	5.91	0.015*	0.013
Trait Reactance	Negative Cognitions	7.09	(1, 499)	9.09	0.003**	0.019
	Anger	29.90	(1, 499)	50.96	< 0.001***	0.096
	Threat to Freedom	28.36	(1, 499)	51.94	< 0.001***	0.098
Strength of Preference	Negative Cognitions	0.92	(1, 499)	1.18	0.279	0.002
	Anger	3.41	(1, 499)	5.82	0.016*	0.012
	Threat to Freedom	0.21	(1, 499)	0.38	0.538	0.001
Mode	Negative Cognitions	1.11	(1, 499)	1.42	0.234	0.003
	Anger	2.14	(1, 499)	3.65	0.057	0.008
	Threat to Freedom	4.50	(1, 499)	8.24	0.004**	0.017
Age	Negative Cognitions	2.18	(1, 499)	2.79	0.095	0.006
	Anger	6.61	(1, 499)	11.26	0.001**	0.023
	Threat to Freedom	1.15	(1, 499)	2.10	0.147	0.004
Gender	Negative Cognitions	0.03	(1, 499)	0.04	0.842	0.000
	Anger	0.02	(1, 499)	0.03	0.856	0.000
	Threat to Freedom	0.20	(1, 499)	0.37	0.543	0.001
Education	Negative Cognitions	0.28	(1, 499)	0.36	0.551	0.001
	Anger	0.66	(1, 499)	1.13	0.289	0.002
	Threat to Freedom	4.77	(1, 499)	8.73	0.003**	0.018
Residuals	Negative Cognitions	372.79	478			
	Anger	280.50	478			
	Threat to Freedom	260.99	478			

^a^SS = Sum of Squares

*p < .05

**p < .01

***p < .001

### Effect on behavioral intentions

In addition, we examined the effect of our experimental manipulations on Behavioral Intention. As shown in Table 2, behavioral intention was significantly negatively correlated with all three reactance measures. This suggests that the more reactance experienced by the participant, the less likely they were to accept the temperature suggestion from the thermostat. We conducted a three-way ANCOVA using Language, Temperature, and Justification as the independent variables and Behavioral Intention as the dependent variable. Trait Reactance and Strength of Preference were included as covariates and we once again controlled for mode, age, gender, and education. The results are summarized in Table 4.

**Table 4:** ANCOVA results for Behavioral Intention.

Measures	SS^a^	df	F	P	*η*_p_^2^
Language	4.27	(3, 497)	1.06	0.366	0.008
Temperature	31.74	(1, 499)	23.61	< 0.001***	0.047
Justification	7.80	(1, 499)	5.80	0.016*	0.012
Language x Temperature	0.92	(3, 497)	0.23	0.876	0.001
Language x Justification	1.49	(3, 497)	0.37	0.774	0.002
Temperature x Justification	8.31	(1, 499)	6.18	0.013*	0.013
Language x Temperature x Justification	3.40	(3, 497)	0.84	0.471	0.005
Trait Reactance	14.84	(1, 499)	11.04	< 0.001***	0.023
Strength of Preference	4.72	(1, 499)	2.77	0.097	0.006
Mode	4.01	(1, 499)	2.98	0.085	0.006
Age	8.13	(1, 499)	6.05	0.014*	0.012
Gender	0.04	(1, 499)	0.03	0.861	0.000
Education	0.16	(1, 499)	0.12	0.730	0.000
Residuals	642.37	478			

^a^SS = Sum of Squares

*p < .05

**p < .01

***p < .001

There was no main effect of Language on Behavioral Intention, suggesting the degree of authoritative language used by SEM did not significantly influence whether participants were willing to accept the temperature adjustments.

However, there was a main effect of Temperature on Behavioral Intention. Participants in the Incongruent condition (M = 3.19, SD = 1.28) were significantly less likely to accept the temperature adjustments than those in the Congruent condition (M = 3.67, SD = 1.12).

There was weak evidence of a main effect of Justification on Behavioral Intention. Participants in the Transparent condition (M = 3.56, SD = 1.13) tended to be more likely to accept the temperature adjustments compared to those in the Intransparent condition (M = 3.30, SD = 1.29).

**Fig 6:**
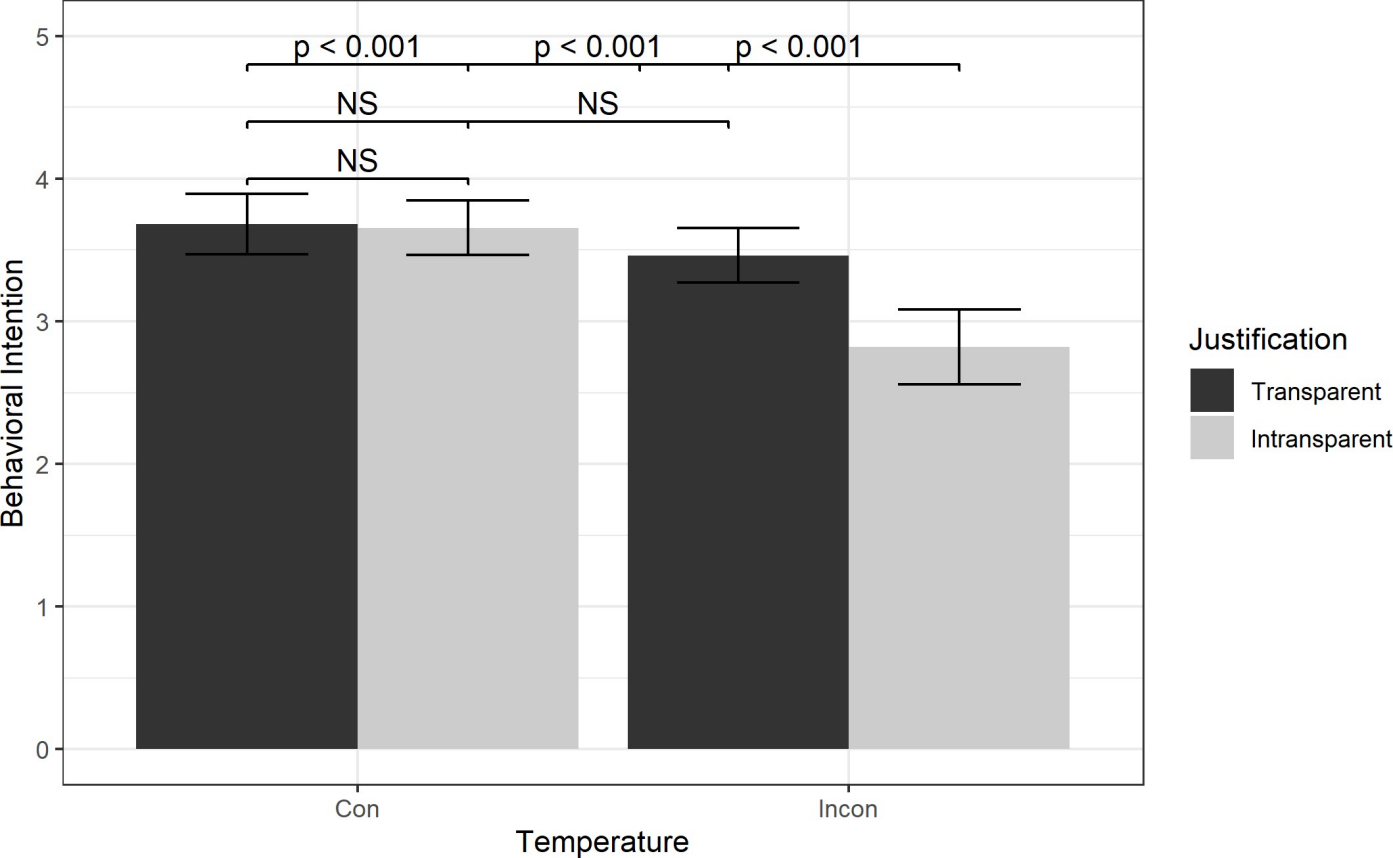
Effect of Temperature and Justification on Behavioral Intention. Results from Tukey post-hoc tests are shown with +/- 2 standard error bars.

There was weak evidence of a significant two-way interaction between Temperature and Justification on Behavioral Intention (see Fig 6). For post-hoc analysis, a Games-Howell test was performed on a one-way ANOVA with the behavioral intention question as the dependent variable and a concatenated Temperature and Justification variable treated as the independent variable. Results indicated participants in the Incongruent condition who did not receive a justification (Intransparent) (M = 2.82, SD = 1.34) were significantly less likely to accept the temperature adjustments than those in the Incongruent condition who received justification (Transparent) (M = 3.46, SD = 1.16, p < 0.001). Those in the Incongruent, Intransparent group were also significantly less likely to accept the temperature adjustments than both the Congruent, Transparent (M = 3.68, SD = 1.10, p < 0.001) and Congruent, Intransparent (M = 3.65, SD = 1.14, p < 0.001) groups. However, participants in the Congruent, Transparent and Congruent, Intransparent were not significantly different from each other (p = 0.998) nor from the Incongruent, Transparent condition (Congruent, Transparent: p = 0.417; Congruent, Intransparent: p = 0.489). This suggests participants were more likely to accept the temperature adjustments if the temperature was within their preferences, regardless of whether a justification was given. Furthermore, even when the temperature was outside their preferences, they were just as likely to accept the adjustment as long as a justification was presented. However, if the temperature was outside user preferences and no justification was given, then the participants were significantly less likely to accept the adjustment compared to the other groups.

**Fig 7:**
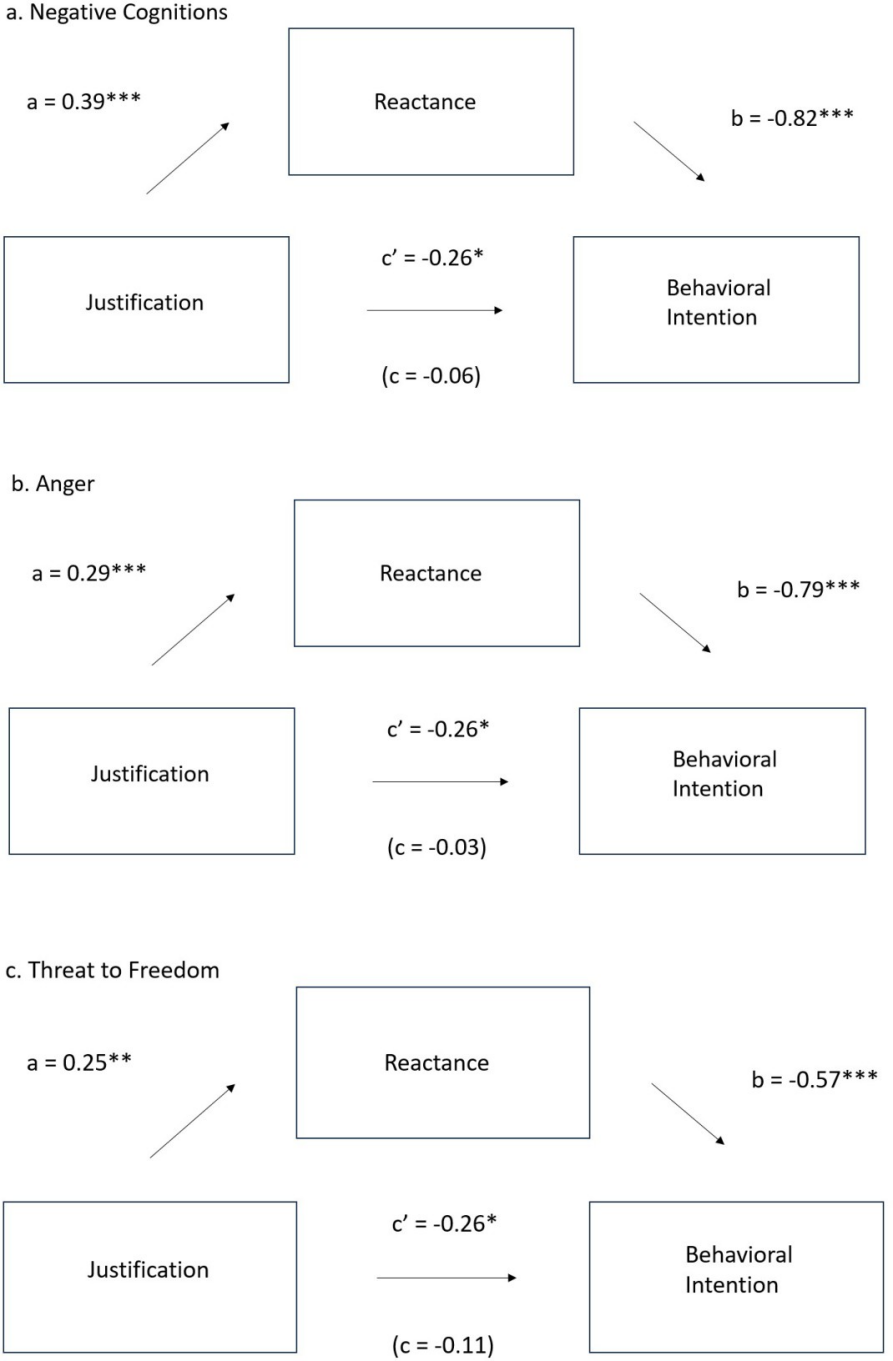
Mediation effects of reactance. This shows the relationship between justification and behavioral intention for (a) negative cognitions, (b) anger, and (c) threat to freedom.

Due to the significant correlations between behavioral intention and the reactance measures, possible mediation effects were examined for the relationship between Justification, the three reactance measures, and Behavioral Intention. To test for mediation, three regression models were created using Behavioral Intention as the outcome variable with Justification and one of the reactance measures as the predictors. The regression analyses revealed the effect of Justification on Behavioral Intention was no longer significant when including the reactance measures in the models. This suggests the relationship between Justification and Behavioral Intention was fully mediated by reactance. As summarized in Fig 7, the regression coefficient between Justification and Behavioral Intention was significant and the regression coefficients between Behavioral Intention and the three reactance measures were significant. The significance of the indirect effect was tested using bootstrapping for 1,000 samples using a 95% confidence interval. The bootstrapped unstandardized effects for Negative Cognitions, Anger, and Threat to Freedom with corresponding confidence intervals were -0.32 (-0.47, -0.18), -0.22 (-0.35, -0.11), and -0.14 (-0.23, -0.05) respectively. Therefore, the indirect effects were significant.

## Discussion

In this study, we found a number of interface design features that induce psychological reactance and may lead a user to override a system’s actions, even if that action comports with the user’s previously stated preferences. We examined three aspects of a smart thermostat that may induce reactance, (1) the degree of authoritative language used in a system notification message about a thermostat setting change (H1), (2) the congruence between the thermostat’s temperature adjustment and the user’s preferences (H2), and (3) whether the thermostat provided a justification for its suggestions (H3). Ultimately, we found support for all three hypotheses. In addition, we validated an experimental paradigm for measuring reactance in an energy context by replicating many results from other domains.

First, we found support for H1 that highly authoritative language will induce reactance and reduce user acceptance. With our manipulation of language, evidence suggested the word “must” induced significantly more reactance than the other language conditions for all three reactance measures. This finding is in line with previous research by Miller (2007) and Roubroeks (2010) [35, 36]. However, this more authoritative language did not lead to greater unwillingness to accept the thermostat’s suggestion.

Second, we found support for H2 that deviating from user-preferred thermostat settings will induce reactance and reduce user acceptance. Deviating from temperature preferences by two degrees had a negative impact. A smart thermostat may need to deviate from user preferences to save energy or reduce the strain on the electrical grid in accordance with the thermostat’s programming. All three measures of reactance were higher when the temperature was incongruent. Participants were also less likely to accept the thermostat change if the temperature was incongruent. This finding is similar to the study by Roubroeks (2010) where participants experienced more reactance when working with a robot washing machine whose goals did not align with their own [36]. In our experiment, participants had a personal preference for temperatures that they find comfortable (i.e., to some degree, they had a goal to be comfortable). When the smart thermostat adjusted the temperature to be outside that range, the goal of the thermostat was not aligned with the goal of the participant.

Third, we found support for H3 that removing a justification will induce reactance and reduce user acceptance. Participants who received a notification about the thermostat change, but did not receive information about current weather conditions, reported more reactance across all three measures when compared to participants who did receive the weather information. Participants who did not receive the weather conditions were also less willing to accept the temperature change. Participants who were given weather conditions may have viewed the temperature adjustments as legitimate and temporary as described by Brehm (1989) and Dillard & Shen (2005) and as such, reported experiencing less reactance than those not provided weather data [31, 39]. Results from Ehrenbrink (2016) support this finding as the robotic entity featured in that study, a smart TV, induced less reactance in users when an explanation was provided with its error messages [41].

Our exploratory analysis suggests that providing weather conditions as a justification was further shown to increase compliance. Participants whose thermostats deviated from their temperature preferences and were not provided weather conditions were less likely to accept the temperature adjustment compared to participants in other groups. However, participants who were given a temperature outside their preferences and were provided the weather conditions were just as likely to accept the temperature adjustment as participants who were given a temperature within their preferences. This is consistent with work by Skraaning and Jamieson (2021) on the automation transparency design principle, which suggests that for some forms of automation, user acceptance of automated actions is influenced by whether “responsibilities, capabilities, goals, activities, and/or effects of automation” are directly observable [56].

Through post-hoc mediation analysis, the effect of justification on behavioral intention was shown to be mediated by the effect of reactance on behavioral intention, suggesting the non-compliant behavior in those not provided weather conditions may have been driven by possible feelings of reactance. This suggests that if it is necessary to ignore a user’s stated setting preferences to achieve a competing goal, providing an explanation may increase compliance. When designing smart thermostats, being transparent with users about why the thermostat is making temperature adjustments is a potential way to persuade users to adopt energy conservation behaviors in their daily lives.

This study has three primary limitations. This study was conducted online and asked participants to imagine interacting with a smart thermostat rather than actually using a device in the home. In a real-world setting the thermostat’s behavior would affect a consumer’s actual autonomy (rather than their hypothetical autonomy), which could affect the experience of reactance differently. Similarly, participants’ willingness to accept the smart thermostat’s temperature change could be affected by the nature of the online experimental set-up since there is no risk of change to their physical comfort. However, participants may also be paying more attention in a laboratory experiment than they would at home.

In addition, future studies should explore the effect of repeated exposure to such a system on inducing reactance and subsequent behavioral compliance. In a repeated exposures context, it would be valuable to see how perceptions of the system change as it makes multiple adjustments over time. To design smart energy management systems that are appealing to consumers and less likely to fall into disuse, it is critical to understand how these systems may induce reactance and to identify strategies for alleviating these feelings.

Finally, because there was no non-anthropomorphic control condition in this study, it is not possible to determine whether anthropomorphism interacts with justification or temperature preferences to affect reactance and/or user acceptance. Previous research has demonstrated an effect of anthropomorphism of robot actors in inducing reactance in human-machine interactions [42, 43]. Therefore, reactance in this study may have been partially mediated by the presence of the anthropomorphic figure, SEM. However, these effects were at least controlled for by having all participants receive the same figure, which is expected to have increased the estimated effect sizes. Future work should explore the individual effect of anthropomorphism on these variables. Manipulating anthropomorphic features, such as the perceived friendliness, of a smart energy management system could affect reactance and compliance when deviating from user preferences.

## Conclusions

When using automated systems and smart technology, users may perceive a threat to their freedoms and thus experience psychological reactance. This psychological reactance can lead to behaviors in the opposite direction of the intended behavior. This is likely to be increasingly important as the use of automated systems increases. When an automated system executes an action for a user, such as turning off the lights or adjusting the thermostat, the system is exerting control over the user, and loss of control is at the heart of inducing psychological reactance. What has not been well researched is whether, in a particular context, this may indeed be perceived as a loss of control by the user, whether this loss of control can be perceived as threatening, and whether this will lead users to reassert control by overriding the system.

Smart thermostats are a common form of automation for consumers looking to use smart energy management systems. Smart thermostats offer the potential to reduce residential energy usage through automation, however, smart thermostats tend to under-deliver due to misuse and disuse [57]. A critical contributing factor in the disuse of Nest thermostats is uncertainty about what the system was doing [19]. By pushing information, requests, and suggestions to the user instead of expecting users to be aware of the dynamic factors influencing the operations of the thermostat, savings can be maximized [21]. However, certain interface design features that might make a system more persuasive, such as social cues and using persuasive language, might also make the system more likely to induce reactance in the user [42].

This study provides insight into design considerations for optimizing smart home energy management systems by focusing on motivational factors. This research has three primary contributions. First, we demonstrate an experimental paradigm where reactance can be observed and manipulated in an energy context. Second, we replicate findings from other domains to validate this experimental paradigm. Third, we find an indirect relationship between reactance and behavioral intentions that is influenced by the presence of a justification. Our results suggest consumers experience less reactance when a smart thermostat uses less authoritative language, does not deviate from temperature preferences, and provides a legitimate explanation for the temperature change. However, experiencing reactance does not always lead to reduced behavioral compliance. Language did not affect behavioral intentions and incongruent temperatures only decreased behavioral intentions when no justification was present. This suggests that energy consumption behavior is not solely driven by emotional reactions such as reactance.

## Supporting information

S1 Table ANCOVA results for interaction effects on reactance. Main effects are in Table 3.
(PDF)
