# Supplementary material for: Don’t touch that dial: Psychological reactance, transparency, and user acceptance of smart thermostat setting changes
Source: PLoS One. 2023 Jul 24;18(7):e0289017. doi: 10.1371/journal.pone.0289017 (PMC10365304; doi:10.1371/journal.pone.0289017)
Supplement: S1 Table — Main effects are in Table 3. (PDF) [file pone.0289017.s001.pdf]

Don’t touch that dial: Psychological reactance, transparency, and user acceptance of smart thermostat setting changes

Supplementary Information

Materials, data, and R code are available at Open Science Framework, https://osf.io/zufmt/.

Table S1. ANCOVA Results for Interaction Effects on Reactance.

Measures	Reactance Measure	SS[a]	df	F	*p*	η_p^2
Language x Temperature	Negative Cognitions	0.71	(3, 497)	0.30	0.823	0.002
	Anger	2.84	(3, 497)	1.62	0.185	0.009
	Threat to Freedom	1.84	(3, 497)	1.12	0.339	0.006
Language x Justification	Negative Cognitions	1.81	(3, 497)	0.78	0.508	0.005
	Anger	0.84	(3, 497)	0.48	0.699	0.004
	Threat to Freedom	1.85	(3, 497)	1.13	0.336	0.007
Temperature x Justification	Negative Cognitions	1.52	(1, 499)	1.95	0.163	0.004
	Anger	0.01	(1, 499)	0.01	0.918	0.000
	Threat to Freedom	0.22	(1, 499)	0.40	0.530	0.001
Language x Temperature x Justification	Negative Cognitions	1.05	(3, 497)	0.45	0.718	0.003
	Anger	3.38	(3, 497)	1.92	0.126	0.012
	Threat to Freedom	1.19	(3, 497)	0.72	0.538	0.005

Main effects are in Table 3.
[a]SS = Sum of Squares
